# Large Bowel Obstruction From a Calcified Uterine Fibroid: A Rare Cause of Extrinsic Compression

**DOI:** 10.7759/cureus.104219

**Published:** 2026-02-25

**Authors:** Amelia J Cooper, Ken Davey

**Affiliations:** 1 General Surgery, Eastern Health, Melbourne, AUS; 2 General Surgery, Albury Wodonga Health, Albury, AUS

**Keywords:** bowel obstruction, caecal volvulus, large bowel obstruction, leiomyoma, uterine fibroid

## Abstract

Large bowel obstruction is a surgical emergency most commonly caused by colorectal malignancy, volvulus, or diverticular disease. Extrinsic compression of the colon from gynaecological pathology is rare, and bowel obstruction secondary to uterine fibroids is exceptionally uncommon. We present the rare case of a 72-year-old postmenopausal woman who presented with acute intermittent abdominal pain, vomiting, and obstipation. Computed tomography demonstrated calcified masses adjacent to the ascending colon with proximal bowel dilatation, consistent with large bowel obstruction. A laparotomy was performed by the general surgery team, and a large calcified exophytic uterine fibroid was found to be compressing the caecum and causing secondary partial caecal volvulus. The obstruction was relieved, and myomectomy was performed after consultation with the gynaecology team. Histopathological examination confirmed a benign leiomyoma with extensive calcification. The patient made an uncomplicated recovery and was discharged on postoperative day four. This case highlights an unusual cause of large bowel obstruction and underscores the importance of considering gynaecological pathology in the differential diagnosis of atypical obstructive presentations, particularly in postmenopausal patients.

## Introduction

Large bowel obstruction (LBO) is a surgical emergency associated with significant morbidity and mortality, most commonly caused by colorectal malignancy, volvulus, or diverticular disease [1]. LBO accounts for approximately 20%-25% of all cases of intestinal obstruction [2] and is associated with reported mortality rates of around 10%-20%, particularly when complicated by bowel ischaemia or perforation [3,4]. Therefore, prompt diagnosis and early surgical intervention are essential to prevent adverse outcomes. Less commonly, extrinsic compression from adjacent pelvic pathology may lead to obstruction, presenting a diagnostic challenge, particularly when imaging findings are atypical.

Uterine fibroids (leiomyomas) are the most common benign tumours in women and typically present with gynaecological symptoms such as abnormal uterine bleeding or pelvic pressure [5]. In postmenopausal women, fibroids generally regress, and new or enlarging fibroids are uncommon [6]. Although fibroids are common, gastrointestinal complications are rare, and bowel obstruction secondary to fibroids is exceptionally uncommon, with only isolated cases reported in the literature [7,8].

We present a rare case of acute LBO caused by an extensively calcified, exophytic uterine fibroid in a postmenopausal woman, complicated by partial caecal volvulus. This case highlights the importance of considering pathology arising from adjacent organ systems, including gynaecological causes, in the differential diagnosis of bowel obstruction and demonstrates the value of multidisciplinary surgical management in atypical presentations.

## Case presentation

A 72-year-old woman presented to a regional emergency department and was admitted under the general surgical unit with 12 hours of suprapubic pain that was severe and intermittent. This was associated with nausea and three episodes of faeculent vomiting. She had not passed flatus or opened her bowels since the onset of the pain. She had chills but no sweats and no urinary symptoms. In the preceding weeks to months, she had no night sweats or unexplained weight loss. She had a history of hypertension, had no previous abdominal surgery, and was fit and independent from home.

On examination, the patient was haemodynamically stable and afebrile and had a mildly distended abdomen with tenderness and guarding in the right iliac fossa and mild suprapubic tenderness. No masses were palpable in the abdomen.

Laboratory studies on admission revealed inflammatory markers, including white cell count and C-reactive protein within normal limits, and a raised lactate (Table 1). After 1 L of crystalloid intravenous fluids, lactate normalised to 1.4 mmol/L.

**Table 1:** Laboratory studies on admission

Parameters	Value	Reference range
Haemoglobin (g/L)	143	115-165
White cell count (x10^9^/L)	8.5	4.0-11.0
C-reactive protein (mg/L)	4	<10
Lactate (mmol/L)	3.8	<2.2

**Figure 1:**
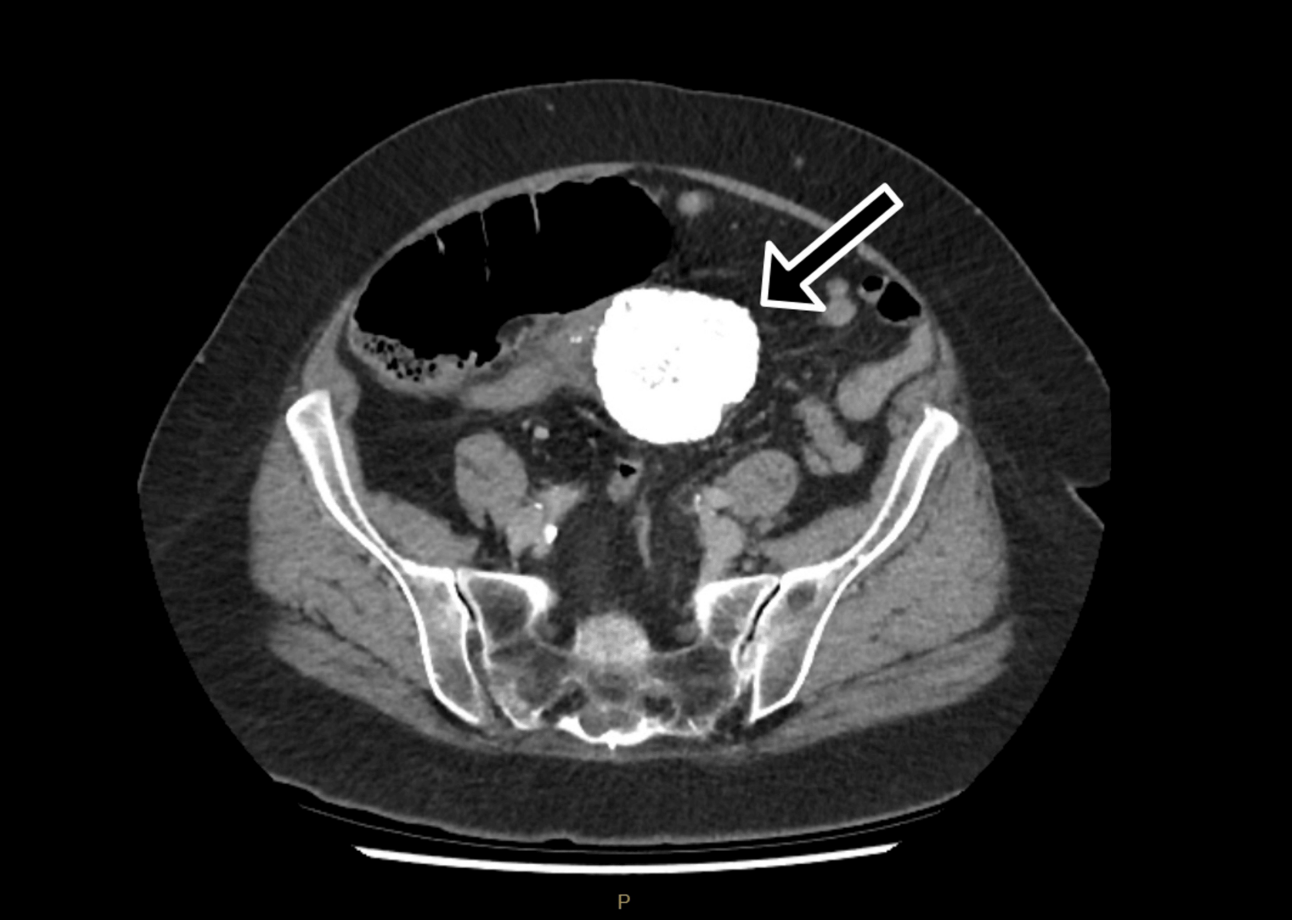
Axial portal venous phase CT of the abdomen demonstrating a large calcified central abdominal mass (arrow) causing proximal colonic dilatation consistent with large bowel obstruction CT: computed tomography

**Figure 2:**
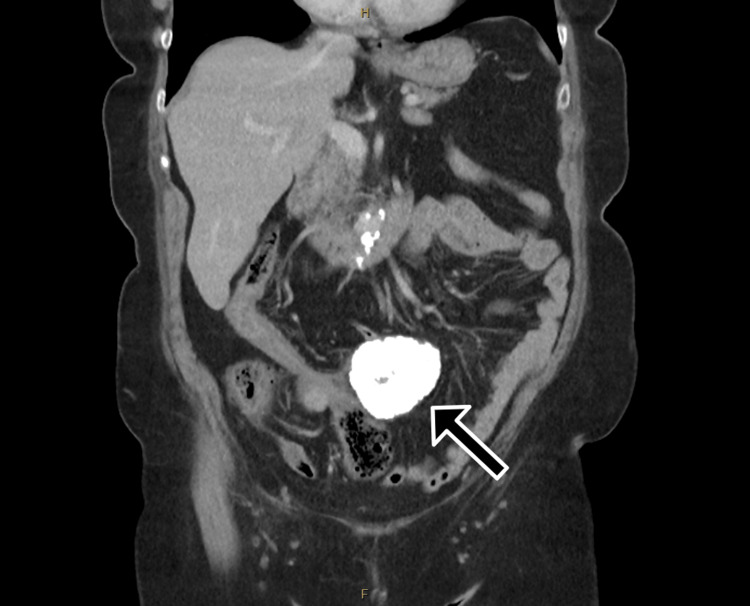
Coronal portal venous phase CT image demonstrating a large calcified mass (arrow) within the central abdomen, with proximal colonic dilatation and distal large bowel collapse consistent with mechanical large bowel obstruction CT: computed tomography

Contrast-enhanced computed tomography (CT) of the abdomen and pelvis demonstrated two calcified masses within the lower abdomen, adjacent to the ascending colon, measuring 65 and 26 mm in maximal diameter (Figures 1, 2). These masses were associated with proximal colonic dilatation with associated small bowel dilatation, with collapse of the colon distal to the lesions, consistent with LBO. A small volume of free fluid was present adjacent to the caecal pole, with no evidence of free gas or intra-abdominal collection. No lymphadenopathy or distant metastatic disease was identified.

Review of prior imaging revealed a lumbar spine radiograph performed eight years earlier, which demonstrated a large pelvic calcification reported as likely representing a calcified uterine fibroid. As the patient was asymptomatic at that time, no further investigation was undertaken.

Based on the imaging appearance and correlation with prior studies, a calcified uterine fibroid was suspected preoperatively. Differential considerations included a calcified mesenteric mass, colonic neoplasm, or other pelvic soft tissue tumours. However, the degree of caecal obstruction and associated volvulus were not fully appreciated until intraoperative assessment.

The patient underwent a laparotomy, which revealed a large calcified mass with exophytic growth arising from the uterine fundus, which had caused obstruction at the caecum with partial caecal volvulus around the mass. The obstruction was reduced intraoperatively; however, a constriction point with some erythema was noted on the caecum. The caecum appeared viable with no evidence of ischaemia, and bowel resection was not required. The remainder of the bowel and the other organs appeared normal. The gynaecology team was consulted intraoperatively, and a myomectomy was recommended. This was performed without complication. The postoperative course was complicated by a transient ileus lasting 48 hours, which resolved with conservative management. The patient was discharged home on postoperative day four.

**Figure 3:**
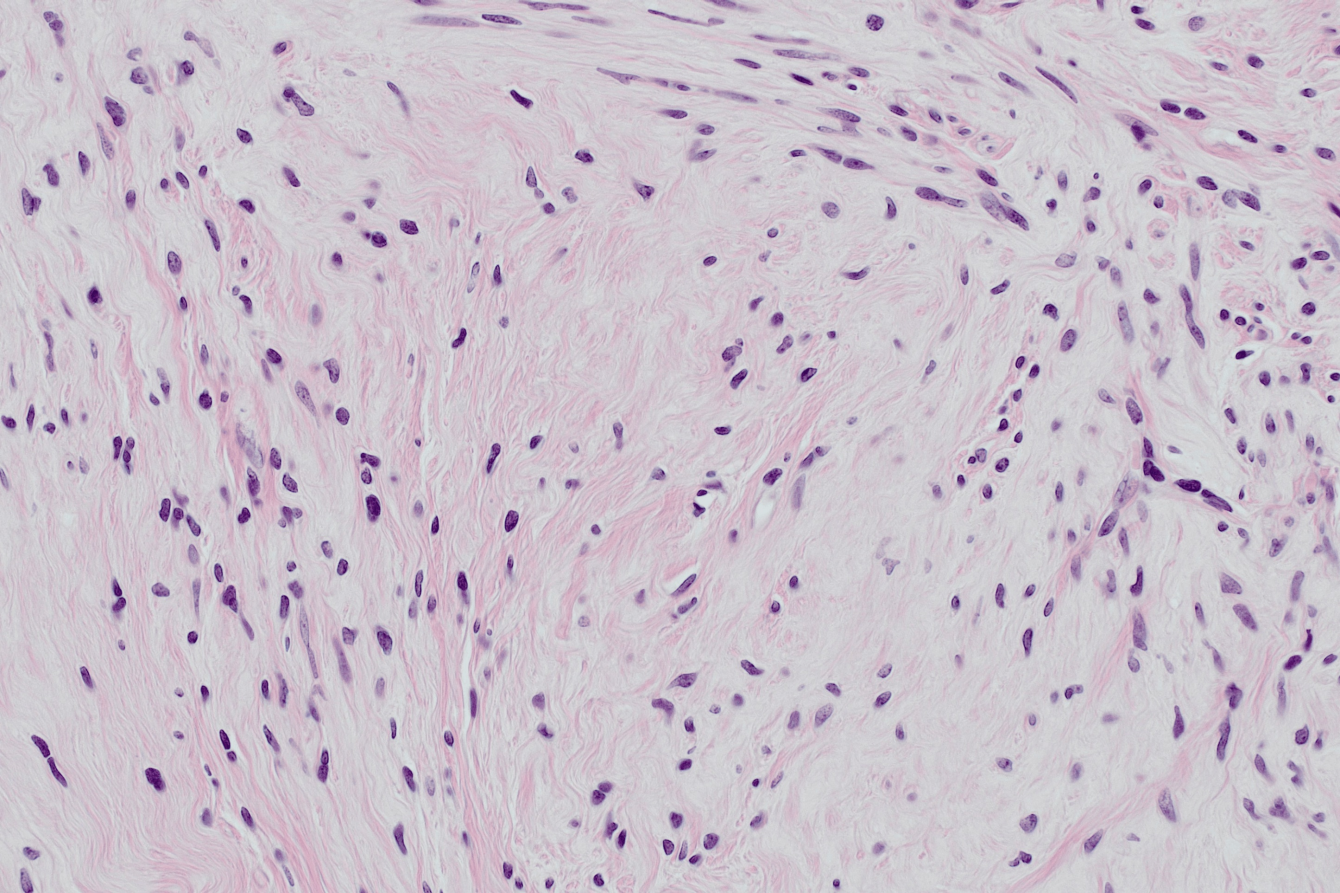
Haematoxylin and eosin-stained section of excised uterine mass demonstrating interweaving fascicles of bland spindle cells without atypia or mitotic activity, with prominent calcification, consistent with leiomyoma (original magnification x200)

Histological examination of the excised uterine mass demonstrated a nodular proliferation of bland spindle cells arranged in interweaving fascicles, without atypia, necrosis, or significant mitotic activity (Figure 3). Extensive areas of calcification were present. Immunohistochemistry showed positivity for actin and desmin and negative staining for AE1/3, supporting smooth muscle differentiation. These findings were consistent with a leiomyoma with prominent calcification.

## Discussion

Bowel obstruction results from mechanical or functional impairment of intestinal transit and may be caused by intraluminal, intrinsic, or extrinsic pathology. Mechanical obstruction leads to proximal bowel dilatation, fluid sequestration, and bacterial overgrowth, with progression to ischaemia, perforation, and sepsis if untreated. The underlying aetiology differs by anatomical location: small bowel obstruction is most commonly caused by postoperative adhesions, whereas LBO is most frequently due to malignancy, followed by volvulus and diverticular disease [9]. Extrinsic compression from pelvic pathology is rare but should be considered when imaging demonstrates atypical masses adjacent to the colon.

Uterine fibroids (leiomyomas) are the most common benign tumours in women, with a lifetime prevalence approaching 70%-80% by the age of 50. They arise from monoclonal proliferation of myometrial smooth muscle cells and are hormone-dependent, typically regressing after menopause [10]. Long-standing fibroids may undergo degenerative change, including hyaline degeneration and calcification, which is more frequently seen in postmenopausal patients. Fibroids may be intramural, submucosal, or subserosal, and exophytic subserosal fibroids can exert mass effect on adjacent pelvic organs, including the bladder, ureters, and bowel [11]. In rare cases, large or pedunculated fibroids may cause extrinsic compression, displacement, or torsion of adjacent bowel segments, leading to mechanical obstruction or volvulus as in this case.

Though frequently asymptomatic, uterine fibroids can cause significant morbidity, including menorrhagia, anaemia, and pregnancy loss [10]. Gastrointestinal involvement of uterine fibroids is uncommon. When bowel symptoms occur, they are usually limited to constipation or altered bowel habit. Uterine fibroids have been known to cause bowel obstruction; however, it is extremely rare, with only isolated case reports documented in medical literature. One such case documented a small bowel obstruction from a large fibroid causing compression of the small bowel [7]. Another documents a case of LBO from a uterine fibroid in the immediate postpartum period following a caesarean section [8]. Compared with previously reported cases, the present case is unusual due to the patient’s postmenopausal status, absence of gynaecological symptoms, and the presence of extensive calcification suggesting long-standing disease.

In this case, an exophytic, heavily calcified fibroid arising from the uterine fundus caused extrinsic compression of the caecum, resulting in LBO with secondary partial caecal volvulus. The patient’s postmenopausal status, absence of gynaecological symptoms, and atypical CT appearance initially raised concern for a primary mesenteric or colonic malignancy. This highlights the diagnostic challenge posed by degenerative fibroids and reinforces the importance of maintaining a broad differential diagnosis for bowel obstruction, particularly when imaging demonstrates calcified pelvic masses.

Management of LBO differs from small bowel obstruction, as conservative management is rarely successful and delays increase the risk of perforation and mortality [12]. Early surgical intervention is therefore recommended. In this case, operative exploration allowed definitive diagnosis, reduction of the volvulus, and curative treatment via myomectomy without bowel resection. Multidisciplinary collaboration between general surgery and gynaecology was essential to achieving an optimal outcome. This case highlights a rare but important cause of LBO and emphasises the need to consider gynaecological pathology in the differential diagnosis of bowel obstruction, even in postmenopausal women.

## Conclusions

This case describes a rare cause of LBO due to extrinsic compression from a calcified uterine fibroid, resulting in secondary caecal volvulus. Although uterine fibroids are common, their presentation with acute bowel obstruction is exceptional, particularly in postmenopausal patients and in the absence of gynaecological symptoms.

The atypical clinical presentation and unusual imaging appearance posed a diagnostic challenge, highlighting the importance of considering pathology arising from adjacent organ systems when evaluating bowel obstruction. Early operative intervention and multidisciplinary collaboration between general surgery and gynaecology were essential to achieving a definitive diagnosis and successful outcome. Awareness of this rare entity may facilitate timely management and prevent delays in treatment in future cases.
